# Breast cancer screening disparities among immigrant women by world region of origin: a population‐based study in Ontario, Canada

**DOI:** 10.1002/cam4.700

**Published:** 2016-04-22

**Authors:** Mandana Vahabi, Aisha Lofters, Matthew Kumar, Richard H. Glazier

**Affiliations:** ^1^ Faculty of Community ServicesDaphne Cockwell School of NursingRyerson UniversityTorontoOntarioCanada; ^2^ Graduate Program in Immigration and Settlement StudiesRyerson UniversityTorontoOntarioCanada; ^3^ Ryerson Centre for Global Health and Health EquityTorontoOntarioCanada; ^4^ Centre for Research on Inner City HealthLi Ka Shing Knowledge InstituteSt. Michael's HospitalTorontoOntarioCanada; ^5^ Department of Family and Community MedicineUniversity of TorontoTorontoOntarioCanada; ^6^ Department of Family and Community MedicineSt. Michael HospitalTorontoOntarioCanada; ^7^ Institute for Clinical Evaluative SciencesTorontoOntarioCanada; ^8^ Dalla, Lana School of Public HealthUniversity of TorontoTorontoOntarioCanada

**Keywords:** Breast cancer, immigrants, immigration class, internationally trained physicians, primary care patient enrollment models, screening mammography, world regions of origin

## Abstract

Rates of mammography screening for breast cancer are disproportionately low in certain subgroups including low‐income and immigrant women. The purpose of the study was to examine differences in rates of appropriate breast cancer screening (i.e., screening mammography every 2 years) among Ontario immigrant women by world region of origin and explore the association between appropriate breast cancer screening among these women groups and individual and structural factors. A cohort of 183,332 screening‐eligible immigrant women living in Ontario between 2010 and 2012 was created from linked databases and classified into eight world regions of origin. Appropriate screening rates were calculated for each region by age group and selected sociodemographic, immigration, and healthcare‐related characteristics. The association between appropriate screening across the eight regions of origin and selected sociodemographic, immigration, and health‐related characteristics was explored using multivariate Poisson regression. Screening varied by region of origin, with South Asian women (48.5%) having the lowest and Caribbean and Latin American women (63.7%) the highest cancer screening rates. Factors significantly associated with lower screening across the world regions of origin included living in the lowest income neighborhoods, having a refugee status, being a new immigrant, not having a regular physical examination, not being enrolled in a primary care patient enrollment model, having a male physician, and having an internationally trained physician. Multiple interventions entailing cross‐sector collaboration, promotion of patient enrollment models, community engagement, comprehensive and intensive outreach to women, and knowledge translation and transfer to physicians should be considered to address screening disparities among immigrant population. Consideration should be given to design and delivery of culturally appropriate and easily accessible cancer screening programs targeted at high‐ risk immigrant subgroups, such as women of South Asian origin, refugees, and new immigrants.

## Introduction

Screening with mammography has been identified as one of the most effective methods for early detection and treatment of breast cancer and has been recommended as part of the Canadian breast cancer screening guidelines since the late 20th century 1. In Canada, it is recommended that women between the ages of 50 and 69 undergo screening mammography every 2 years 1. Breast cancer is the second leading cause of cancer deaths among Canadian women. In 2014, there were 24,400 new cases and 5000 deaths due to breast cancer in Canada 2. While rates of mammography have been increasing considerably over the past few decades, screening participation in Ontario is currently at 61% 3, lower than the Cancer Care Ontario, and national targets of 70% 3, 4. In addition, certain subgroups including low‐income and immigrant women have been overrepresented among those under and never screened for cancer 3, 4, 5, 6, 7. A multitude of personal‐, provider‐, and system‐level barriers (e.g., limited health literacy, unfamiliarity with the healthcare system and healthcare entitlements, language difficulty, cultural beliefs and values, limited social support, inadequate financial power, restricted transportation, not having a family physician, racism, and discrimination) have been reported as contributing factors to the low utilization of screening mammography among immigrant women 5, 8, 9, 10, 11, 12, 13, 14. However, it is still unclear what specific immigrant subgroups are most reticent to undergoing screening. Studies exploring breast cancer screening utilization by immigrant women often consider them as a homogenous group and fail to account for the diversity that exists among this population. Immigrants are a heterogeneous group consisting of not only diverse ethnic, cultural, and religious affiliations, but also trajectories of acculturation that are based on the circumstances of their immigration (e.g., immigration class). These factors in turn can heavily influence immigrant women's health, health behaviors, and healthcare utilization. This is particularly relevant in Canada where there is a high concentration of immigrants from many regions of the world. According to the 2011 National Health Survey, 6.8 million Canadians were foreign‐born contributing to 21% of the population, the highest proportion among the eight leading industrial and developed countries in the world 15, 16, 17. The majority of immigrants (53%) in Canada live in Ontario. The source countries for immigrants residing in Canada have been changing over time. During the period between 2006 and 2011, Canada experienced an increased share of immigrants arriving from Asia, Africa, the Caribbean, and South and Central America. In 2013, ~62% of immigrants entered Canada under the economic class, 27% family class, and 11% in the refugee/humanitarian class. The immigration class configurations diverge considerably depending on the region of immigration 17. Although it is plausible that the region of immigration as well as immigration class may play a role in women's utilization of breast cancer screening services, this concern has not been fully explored. Only a few studies reported notable differences in immigrant women's breast and cervical cancer screening by their region of origin 6, 18, 19. Thus, it is imperative to ascertain the specific subgroups of immigrants that are highly vulnerable to being underscreened and highlight the related contributing factors in order to develop and implement culturally appropriate strategies which could encourage cancer screening and reduce cancer disparities.

The objectives of this study were (1) to examine differences in rates of appropriate breast cancer screening (screening mammography every 2 years) among Ontario immigrant women by world region of origin, (2) to explore the association between appropriate breast cancer screening among immigrant women by their world region of origin and individual and structural factors (e.g., age, neighborhood income, length of stay in Canada, immigration class, comorbidities, primary care physician visits, periodic health examinations, physician's gender and training, and type of primary care patient enrollment model (PEM; see Table 1 for different PEMs in Ontario).

**Table 1:** Description of primary care enrollment models in Ontario and description of databases used in study

Physician model	Description
Family Health Group (FHG)	Includes three or more physicians with patient enrollment and some extended hours (weekday evenings and/or weekends). Fee‐for‐service and some physician incentives and bonuses for enrolled patients
Comprehensive Care Model (CCM)	Solo physicians with some patient enrollment and a few extended hours (weekday evenings and/or weekends). Fee‐for‐service and some physician incentives and bonuses for enrolled patients
Family Health Networks (FHN)	Includes three or more physicians with signed governance, patient enrollment, regulated extended hours (weekday evenings and/or weekends). Blended capitation model with physician incentives and bonuses
Family Health Organizations (FHO)	Group includes three or more physicians with signed governance, patient enrollment, and some regulated extended hours. Blended capitation model plus incentives and bonuses
Family Health Team (FHT)	Interdisciplinary teams with patient enrollment and regular extended hours. Physicians must belong to FHN, FHO, or Blended Salary Model
Other	Includes groups such as Rural Northern Physician Group Agreement Complement‐based remuneration plus bonuses and incentives and Community Health Centres (CHC) (salaried model)
No group	Patient's usual primary care provider is not part of a primary care enrollment model
No care	No primary care billings from a primary care provider

Database	Description
Citizenship and Immigration Canada (CIC)	CIC includes demographic information about individuals' at their entry into Ontario as permanent residents from 1985 to 2010. It excludes temporary residents (e.g., students, foreign workers and refugee claimants, those immigrants who landed after 2010, those who declared to move to another province but instead moved to Ontario, and those who could not be probabilistically linked to other databases
Ontario Cancer Registry (OCR)	OCR captures cancer incidence and mortality information of Ontario residents
Ontario Breast Screening Program (OBSP)	The OBSP is a program of Cancer Care Ontario that provides breast cancer screening for women aged 50 to 74 years. OBSP database is an Integrated Client Management System database that contains administrative, clinical, and demographic data for each client screened in the OBSP
Registered Persons Database (RPDB)	Includes residential and demographic information of all Ontario's residents who are eligible for healthcare coverage. The eligibility includes being Canadian Citizens, landed immigrants, or refugees; their primary and permanent residence is in Ontario; and physically resides in Ontario in any 12‐month period for a minimum of least 153 days. For those born outside Ontario, the healthcare coverage starts 3 months after their residency begins
Ontario Physicians' Claims Database—OHIP Claims	Includes billing and diagnostic information submitted by ~95% of Ontario's physicians
ICES Physician Database (IPDB)	Comprises information from the Ontario Health Insurance Plan (OHIP) about the healthcare providers including demographics (training, year of graduation), specialization, and workload (type of work, place of work, location, payment plan, FTEs)
Canadian Institute for Health Information Discharge Abstract Database (CIHI‐DAD)	Includes acute in‐patient hospital discharge data (i.e., demographic, administrative, and clinical information)
The Client Agency Program Enrolment (CAPE) tables	This is a repository of the association of a registered person with a specific physician at a specific agency in a formally recognized program, including primary care patient enrollment models
OHIP Corporate Provider Database (CPDB)	This is a provider registry which includes providers' demographics and their organizations' characteristics. It also includes providers' credentials from the College of Physicians and Surgeons of Ontario (CPSO)
2006 Canadian Census	The census provides demographic and statistical data for all people living in Canada

More details about the physician models can be found at: http://www.healthforceontario.ca/en/Home/Physicians/Training_%7C_Practising_in_Ontario/Physician_Roles/Family_Practice_Models.

## Methods

### Data sources

The study included analysis of several linked population‐level administrative health databases to determine prevalence of mammography screening among Ontario women from different regions of origin, and the determinants of appropriate screening. Databases included the following: The Citizenship and Immigration Canada (CIC) database, 2006 Canadian Census; cancer databases: Ontario Cancer Registry (OCR); Ontario Breast Screening Program (OBSP); and health service utilization databases: Registered Persons Database (RPDB); Ontario Physicians' Claims Database—Ontario Health Insurance Plan (OHIP) Claims; Institute for Clinical Evaluative Sciences (ICES) Physician Database (IPDB); Canadian Institute for Health Information Discharge Abstract Database (CIHI‐DAD), The Client Agency Program Enrolment (CAPE) tables; the OHIP Corporate Provider Database (CPDB). More details related to these databases are given in Table 1. These datasets were linked using unique, encoded identifiers and analyzed at the ICES.

Research ethics approval was obtained from the Sunnybrook Health Sciences Centre Research Ethics Board. All personal identifiers (except for birth year, registration date with the health insurance plan, area of residence, and an encoded unique identifier) were removed from the dataset.

### Inclusion/exclusion criteria

The study included identified immigrants who were captured in the CIC data (i.e., had arrived in Canada between 1985 and 2010). A study cohort was defined using the RPDB that included women aged 50–69 years who were alive and eligible for Ontario's universal health coverage from 1 April 2010 to 31 March 2012, and lived in an Ontario Census Metropolitan Area (CMA) (includes ~82% of Ontario's population) 20. Ontario provincial guidelines recommend mammography screening every 2 years, hence the 2‐year study period was selected. There were a total of 187,410 women who were identified as immigrants from the CIC data, who were eligible for health coverage, and who were aged 50–69 years as of 31 March 2012. Of those, 184,497 lived in CMAs (98.4%). Further exclusions included 4048 women because of prior breast cancer before or on 1 April 2010, and a further 30 due to mastectomy, axillary lymph node, or prophylactic ovary removal before or on 31 March 2012 as their mammography could have been for diagnostic rather than screening purposes. Thus, the study cohort included a total of 183,332 immigrant women (61.8% were aged 50–59 and 38.2% aged 60–69).

The women were classified into eight regions of origin based on their country of birth as obtained from the CIC database. A modified classification based on the World Bank system was used to group the countries into eight regions (1, Caribbean and Latin America; 2, East Asia and Pacific; 3, Eastern Europe and Central Asia; 4, Middle East and North Africa; 5, South Asia; 6, Sub‐Saharan Africa; 7, USA, Australia, and New Zealand; and 8, Western Europe) 18, 21.

### Outcome measure

The main outcome measure was dichotomous: whether a woman had received appropriate screening for breast cancer (i.e., at least one mammogram in the 2‐year study period).

### Statistical analysis

A comparison of the baseline characteristics (including sociodemographic, immigration, and health‐related characteristics) was conducted across the eight regions of origin by age group (i.e., 50–59, 60–69, and overall 50–69 years). Screening rates were then calculated for the three age groups by region of origin and by length of stay in Canada. The following three groups were used for length of stay: women who had been in Canada 5 years or less (*new immigrants*)*,* 6–10 years (*recent immigrants*), and 11 years or more (*established immigrants*). The association between appropriate screening across the eight groups and selected sociodemographic, immigration, and health‐related characteristics was explored using multivariate Poisson regression, because the outcome is common 22. The variables included in the regression were neighborhood income (lower four quintiles vs. highest quintile), resource utilization bands (RUB) (i.e., expected healthcare costs) 23 (RUB 2, 3, 4/5 vs. RUB 1), having a periodic health examination versus not having one, type of primary care PEM (Family Health Group [FHG]/Comprehensive Care Model [CCM], Family Health Networks [FHN]/Family Health Organizations [FHO], No group, Others vs. Family Health Team [FHT]), physician gender (male vs. female), domestically versus internationally trained family physician, urban versus rural residence, immigrant class (family, refugee vs. economic), and length of stay in Canada (new and recent vs. established immigrants).

## Results

### Baseline characteristics

Table 2 presents baseline sociodemographic, immigration, and health‐related characteristics of the study cohort by region of origin. Of the 183,332 immigrant women, the highest proportion came from East Asia and Pacific (28.8%) followed by South Asia (21.1%). The smallest proportion was from USA, Australia, and New Zealand (2.1%). For all immigrant groups, women were mainly in the younger age group. There was considerable variation in sociodemographic characteristics by region of origin. Caribbean, Latin America, and Sub‐Saharan African women were most likely to live in low‐income neighborhoods. There were differences in immigration class (Economy, Family, and Refugees) across region of origin, and this varied by age group, with higher proportions of the older women in most groups having arrived under the Family class. Sub‐Saharan Africa and Eastern Europe had the highest proportion of refugees. The majority of women were established immigrants with Western Europe (88.2%) having the highest proportion, while Middle East and North Africa (13.0%) had the highest proportion of new immigrants.

**Table 2:** Baseline sociodemographic and immigration‐related characteristics of the 183,332 immigrant women (CIC) aged 50–69 in the study population who lived in Ontario's metropolitan areas for the study period 1 April 2010 to 31 March 2012, by region of origin

Value	Caribbean and Latin America	East Asia and Pacific	Eastern Europe and Central Asia	Middle East and North Africa	South Asia	Sub Saharan Africa	USA, Australia and New Zealand	Western Europe	Total
Sociodemographic factors									
*N* (%)	28,071 (15.3)	52,738 (28.8)	29,317 (16.0)	13,058 (7.1)	38,733 (21.1)	7774 (4.2)	3913 (2.1)	9728 (5.3)	183,332 (100)
Mean age ± SD	58.38 ± 4.94	58.23 ± 4.91	58.05 ± 4.68	58.58 ± 5.10	59.40 ± 5.14	57.71 ± 4.86	58.05 ± 4.72	58.21 ± 4.91	58.47 ± 4.96
Median age (IQR)	57 (54–62)	57 (54–62)	57 (54–61)	58 (54–63)	59 (55–64)	56 (54–61)	57 (54–61)	57 (54–62)	57 (54–62)
Income quintile									
1	9755 (34.8%)	12,649 (24.0%)	6982 (23.8%)	2689 (20.6%)	9708 (25.1%)	2757 (35.5%)	549 (14.0%)	1697 (17.4%)	46,786 (25.5%)
2	7067 (25.2%)	13,199 (25.0%)	5561 (19.0%)	2287 (17.5%)	10,055 (26.0%)	1557 (20.0%)	632 (16.2%)	2118 (21.8%)	42,476 (23.2%)
3	5520 (19.7%)	10,573 (20.0%)	5819 (19.8%)	2714 (20.8%)	9555 (24.7%)	1279 (16.5%)	694 (17.7%)	1854 (19.1%)	38,008 (20.7%)
4	3638 (13.0%)	9514 (18.0%)	6639 (22.6%)	2927 (22.4%)	6402 (16.5%)	1219 (15.7%)	774 (19.8%)	1870 (19.2%)	32,983 (18.0%)
5	2055 (7.3%)	6617 (12.5%)	4272 (14.6%)	2422 (18.5%)	2981 (7.7%)	950 (12.2%)	1253 (32.0%)	2172 (22.3%)	22,722 (12.4%)
Missing	36 (0.1%)	186 (0.4%)	44 (0.2%)	19 (0.1%)	32 (0.1%)	12 (0.2%)	11 (0.3%)	17 (0.2%)	357 (0.2%)
Rural									
No	27,845 (99.2%)	52,430 (99.4%)	28,933 (98.7%)	13,020 (99.7%)	38,628 (99.7%)	7710 (99.2%)	3303 (84.4%)	8669 (89.1%)	180,538 (98.5%)
Yes	218 (0.8%)	287 (0.5%)	379 (1.3%)	36 (0.3%)	97 (0.3%)	61 (0.8%)	608 (15.5%)	1054 (10.8%)	2740 (1.5%)
Missing	8 (0.0%)	21 (0.0%)	≤5 (0.0%)	≤5 (0.0%)	8 (0.0%)	≤5 (0.0%)	≤5 (0.1%)	≤5 (0.1%)	54 (0.0%)
Immigration class									
Economic	10,863 (38.7%)	33,022 (62.6%)	10,312 (35.2%)	6231 (47.7%)	12,424 (32.1%)	3268 (42.0%)	1149 (29.4%)	6567 (67.5%)	83,836 (45.7%)
Family	12,612 (44.9%)	15,744 (29.9%)	9736 (33.2%)	3315 (25.4%)	19,904 (51.4%)	1633 (21.0%)	2584 (66.0%)	2642 (27.2%)	68,170 (37.2%)
Other^a^	819 (2.9%)	1204 (2.3%)	552 (1.9%)	191 (1.5%)	512 (1.3%)	188 (2.4%)	179 (4.6%)	180 (1.9%)	3825 (2.1%)
Refugees	3777 (13.5%)	2768 (5.2%)	8717 (29.7%)	3321 (25.4%)	5893 (15.2%)	2685 (34.5%)	≤5 (0.0%)	339 (3.5%)	27,501 (15.0%)
Length of stay in Canada									
Established	22,653 (80.7%)	41,484 (78.7%)	24,298 (82.9%)	8747 (67.0%)	24,517 (63.3%)	5980 (76.9%)	2819 (72.0%)	8577 (88.2%)	139,075 (75.9%)
Recent	3489 (12.4%)	7539 (14.3%)	3486 (11.9%)	2617 (20.0%)	9684 (25.0%)	1198 (15.4%)	694 (17.7%)	730 (7.5%)	29,437 (16.1%)
New	1929 (6.9%)	3715 (7.0%)	1533 (5.2%)	1694 (13.0%)	4532 (11.7%)	596 (7.7%)	400 (10.2%)	421 (4.3%)	14,820 (8.1%)
Number of years in Canada									
Mean ± SD	17.52 ± 6.83	16.29 ± 6.34	16.85 ± 6.13	14.15 ± 6.80	13.03 ± 6.18	16.27 ± 6.75	16.17 ± 7.62	19.80 ± 6.29	15.91 ± 6.69
Median (IQR)	19 (12–23)	18 (11–21)	18 (12–22)	15 (8–20)	12 (8–18)	18 (11–22)	17 (9–23)	22 (17–24)	17 (10–21)
Healthcare‐related factors									
RUBs^b^ (categorized)									
0–1	1577 (5.6%)	4797 (9.1%)	2551 (8.7%)	1090 (8.3%)	2817 (7.3%)	611 (7.9%)	538 (13.7%)	1002 (10.3%)	14,983 (8.2%)
2	2401 (8.6%)	6724 (12.7%)	3374 (11.5%)	1059 (8.1%)	3205 (8.3%)	744 (9.6%)	514 (13.1%)	1276 (13.1%)	19,297 (10.5%)
3	17,414 (62.0%)	33,299 (63.1%)	17,509 (59.7%)	7578 (58.0%)	24,768 (63.9%)	4648 (59.8%)	2117 (54.1%)	5673 (58.3%)	113,006 (61.6%)
4+	6679 (23.8%)	7918 (15.0%)	5883 (20.1%)	3331 (25.5%)	7943 (20.5%)	1771 (22.8%)	744 (19.0%)	1777 (18.3%)	36,046 (19.7%)
ADG^c^									
Mean ± SD	6.60 ± 3.61	5.64 ± 3.31	5.93 ± 3.56	6.72 ± 3.84	6.28 ± 3.50	6.40 ± 3.70	5.39 ± 3.72	5.59 ± 3.50	6.07 ± 3.54
Median (IQR)	6 (4–9)	5 (3–8)	6 (3–8)	7 (4–9)	6 (4–9)	6 (4–9)	5 (3–8)	5 (3–8)	6 (3–8)
ADG (categorized)									
0	1121 (4.0%)	3460 (6.6%)	1776 (6.1%)	844 (6.5%)	2262 (5.8%)	465 (6.0%)	406 (10.4%)	658 (6.8%)	10,992 (6.0%)
1–5	10,273 (36.6%)	23,105 (43.8%)	12,238 (41.7%)	4255 (32.6%)	14,264 (36.8%)	2829 (36.4%)	1748 (44.7%)	4432 (45.6%)	73,144 (39.9%)
6–9	10,631 (37.9%)	19,544 (37.1%)	10,484 (35.8%)	4842 (37.1%)	15,223 (39.3%)	2882 (37.1%)	1192 (30.5%)	3272 (33.6%)	68,070 (37.1%)
10+	6046 (21.5%)	6629 (12.6%)	4819 (16.4%)	3117 (23.9%)	6984 (18.0%)	1598 (20.6%)	567 (14.5%)	1366 (14.0%)	31,126 (17.0%)
Physician enrollment model									
FHG/CCM	14,673 (52.3%)	33,702 (63.9%)	13,589 (46.4%)	7280 (55.8%)	25,884 (66.8%)	3932 (50.6%)	1022 (26.1%)	3105 (31.9%)	103,187 (56.3%)
FHO/FHN	7225 (25.7%)	9345 (17.7%)	7369 (25.1%)	3382 (25.9%)	6212 (16.0%)	2097 (27.0%)	1289 (32.9%)	3252 (33.4%)	40,171 (21.9%)
FHT	2013 (7.2%)	2696 (5.1%)	2377 (8.1%)	668 (5.1%)	1354 (3.5%)	633 (8.1%)	923 (23.6%)	1804 (18.5%)	12,468 (6.8%)
No care	992 (3.5%)	1906 (3.6%)	1470 (5.0%)	597 (4.6%)	1613 (4.2%)	446 (5.7%)	316 (8.1%)	474 (4.9%)	7814 (4.3%)
No group	3078 (11.0%)	4738 (9.0%)	4433 (15.1%)	1100 (8.4%)	3558 (9.2%)	621 (8.0%)	228 (5.8%)	966 (9.9%)	18,722 (10.2%)
Other^d^	90 (0.3%)	351 (0.7%)	79 (0.3%)	31 (0.2%)	112 (0.3%)	45 (0.6%)	135 (3.5%)	127 (1.3%)	970 (0.5%)
Physician training									
Domestic	17,399 (62.0%)	32,372 (61.4%)	11,583 (39.5%)	5194 (39.8%)	12,186 (31.5%)	4498 (57.9%)	3004 (76.8%)	6733 (69.2%)	92,969 (50.7%)
International	9485 (33.8%)	18,338 (34.8%)	16,072 (54.8%)	7204 (55.2%)	24,770 (64.0%)	2790 (35.9%)	562 (14.4%)	2451 (25.2%)	81,672 (44.5%)
Missing	1187 (4.2%)	2028 (3.8%)	1662 (5.7%)	660 (5.1%)	1777 (4.6%)	486 (6.3%)	347 (8.9%)	544 (5.6%)	8691 (4.7%)
Physician from same region of origin									
No	25,345 (90.3%)	39,585 (75.1%)	18,524 (63.2%)	7511 (57.5%)	19,442 (50.2%)	7068 (90.9%)	3880 (99.2%)	8599 (88.4%)	129,954 (70.9%)
Yes	2726 (9.7%)	13,153 (24.9%)	10,793 (36.8%)	5547 (42.5%)	19,291 (49.8%)	706 (9.1%)	33 (0.8%)	1129 (11.6%)	53,378 (29.1%)

CIC, Citizenship and Immigration Canada; FHG, Family Health Group; CCM, Comprehensive Care Model; FHO, Family Health Organizations; FHN, Family Health Networks; FHT, Family Health Team.

a Other includes immigrants who are classified as temporary resident permit holders, retirees, category not stated, postdetermination refugee claimants in Canada, humanitarian and compassionate cases, Canadian experience class, deferred removal orders.

b RUB = resource utilization bands are part of the Johns Hopkins Adjusted Clinical Group^®^ (ACG^®^) Case Mix System. The RUBs are used to categorize patients based on their expected use of healthcare resources and range from 0 (lowest expected healthcare costs) to 5 (highest expected healthcare costs).

c ADG = aggregated diagnosis groups are part of the Johns Hopkins Adjusted Clinical Group^®^ (ACG^®^) Case Mix system. The ADGs are used to measure the level of comorbidity and range from 0 (no diagnosis group) to 32 (32 distinct diagnosis groups).

d Other = other primary care enrollment models, for example, Rural Northern Physician Group Agreement and Community Health Centres.

Immigrant women's health‐related characteristics varied by region of origin. The majority of women were enrolled in FHG/CCM except for those from USA, Australia, and New Zealand, and Western Europe where there were higher proportions enrolled in FHN/FHOs and FHTs. Surprisingly, USA, Australia, and New Zealand had the highest proportion (8.1%) with no care, while Caribbean and Latin America had the lowest (3.5%). USA, Australia, and New Zealand followed by Western Europe had the highest proportion of women seeing domestically trained physicians, while South Asia, Middle East and North Africa, and Eastern Europe and Central Asia had the highest proportion of women seeing internationally trained doctors.

### Breast cancer screening rates

#### Screening rates by region of origin

**Figure 1:**
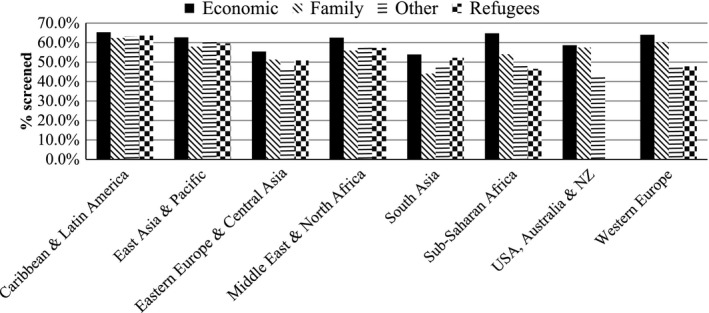
Mammography screening rates of immigrant women by region of origin and immigration class.

**Figure 2:**
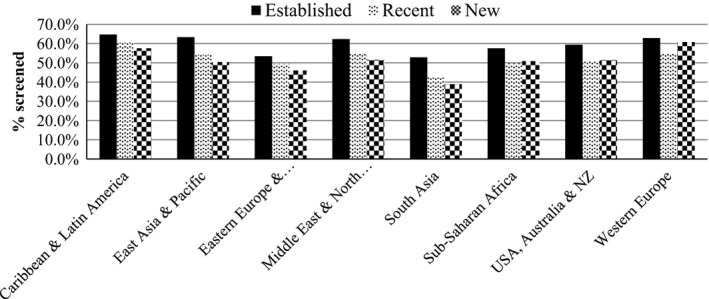
Mammography screening rates of immigrant women by region of origin and length of stay in Canada.

Table 3 shows screening rates of immigrant women by sociodemographic and immigration‐related characteristics. Overall, 57.1% of immigrant women were screened, with slightly lower screening rates for older women (55.0%) compared to younger women (58.4%). South Asian women had the lowest rates (48.5%), followed by Eastern European and Central Asian women (52.5%), while Caribbean and Latin American women (63.7%) and Western European women (62.1%) had the highest rates. Screening rates increased with increasing neighborhood income for all regions of origin; Sub‐Saharan African women had the highest income gradient (19% difference between lowest and highest income) compared to 3.6% difference among Caribbean and Latin American women. Interestingly, Sub‐Sahara African immigrants in the highest income quintile had the highest rate of screening compared to the other groups (67.4%). Generally, rates were highest for women who entered Canada under the Economic class and lowest for the Family or Refugee class. Established immigrants had higher rates than new and recent immigrants (see Figs. 1 and 2). For the majority of region of origin groups, new immigrants (i.e., less than 5 years) had the lowest rates of screening except for Middle East and North and Sub‐Saharan African immigrants, where rates were nearly similar for new and recent immigrants. Overall, among new immigrants, South Asian women had the lowest (38.9%) while Western European women had the highest rates (60.8%).

**Table 3:** Screening rates for immigrant women (CIC) in the study population who lived in Ontario's metropolitan areas for the study period 1 April 2010 to 31 March 2012, by sociodemographic and immigration‐related characteristics and region of origin

Value	Caribbean and Latin America		East Asia and Pacific		Eastern Europe and Central Asia		Middle East and North Africa	
	*N*	%	*N*	%	*N*	%	*N*	%
Age 50–69 years	17,880	63.7	32,212	61.1	15,396	52.5	7765	59.5
Age 50–59 years	11,281	64.2	20,926	62.1	10,228	53.7	4813	60.2
Age 60–69 years	6599	62.8	11,286	59.3	5168	50.4	2952	58.2
Income quintile								
1	6071	62.2	7235	57.2	3372	48.3	1441	53.6
2	4452	63.0	8086	61.3	2841	51.1	1355	59.2
3	3565	64.6	6567	62.1	3133	53.8	1621	59.7
4	2420	66.5	6057	63.7	3651	55.0	1817	62.1
5	1352	65.8	4133	62.5	2375	55.6	1522	62.8
Missing	20	55.6	134	72.0	24	54.5	9	47.4
Rural								
No	17,746	63.7	32,043	61.1	15,203	52.5	7745	59.5
Yes	129	59.2	157	54.7	192	50.7	19	52.8
Missing	≤5	—	12	57.1	≤5	—	≤5	—
Immigration class								
Economic	7093	65.3	20,700	62.7	5712	55.4	3897	62.5
Family	7866	62.4	9124	58.0	4992	51.3	1854	55.9
Other^a^	518	63.2	738	61.3	261	47.3	111	58.1
Refugees	2403	63.6	1650	59.6	4431	50.8	1903	57.3
Length of stay in Canada								
Established	14,659	64.7	26,261	63.3	12,971	53.4	5452	62.3
Recent	2111	60.5	4079	54.1	1720	49.3	1439	55.0
New	1110	57.5	1872	50.4	705	46.0	874	51.6

Value	South Asia		Sub‐Saharan Africa		USA, Australia, and New Zealand		Western Europe	
	*N*	%	*N*	%	*N*	%	*N*	%
Age 50–69 years	18,800	48.5	4341	55.8	2236	57.1	6042	62.1
Age 50–59 years	10,585	50.8	2931	55.5	1479	57.7	3900	62.7
Age 60–69 years	8215	45.9	1410	56.6	757	56.2	2142	61.1
Income quintile								
1	4349	44.8	1329	48.2	277	50.5	976	57.5
2	4862	48.4	866	55.6	337	53.3	1303	61.5
3	4725	49.5	731	57.2	390	56.2	1173	63.3
4	3272	51.1	767	62.9	457	59.0	1205	64.4
5	1575	52.8	640	67.4	770	61.5	1379	63.5
Missing	17	53.1	8	66.7	≤5	—	6	35.3
Rural								
No	18,752	48.5	4300	55.8	1907	57.7	5384	62.1
Yes	42	43.3	40	65.6	328	53.9	656	62.2
Missing	6	75.0	≤5	—	≤5	—	≤5	—
Immigration class								
Economic	6699	53.9	2117	64.8	674	58.7	4204	64.0
Family	8779	44.1	883	54.1	1485	57.5	1590	60.2
Other	242	47.3	91	48.4	77	43.0	86	47.8
Refugees	3080	52.3	1250	46.6	0	—	162	47.8
Length of stay in Canada								
Established	12,954	52.8	3444	57.6	1676	59.5	5387	62.8
Recent	4083	42.2	594	49.6	354	51.0	399	54.7
New	1763	38.9	303	50.8	206	51.5	256	60.8

CIC, Citizenship and Immigration Canada.

a Other includes immigrants who are classified as temporary resident permit holders, retirees, category not stated, postdetermination refugee claimants in Canada, humanitarian and compassionate cases, Canadian experience class, deferred removal orders.

**Figure 3:**
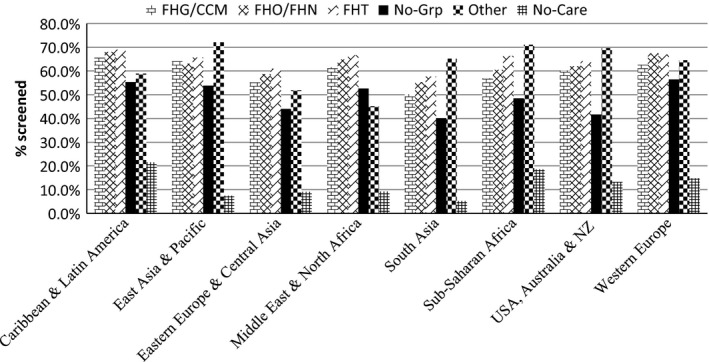
Mammography screening rates of immigrant women by region of origin and primary care model.

Table 4 presents the screening rates for immigrant women by their health‐related characteristics. As expected, screening rates increased with increasing healthcare resource utilization for all regions. There was considerable variation in screening rates by type of physician enrollment model. Overall, screening rates were lowest for those women with no care, with rates ranging from 5.3% for South Asian women to 21.5% for Caribbean and Latin American women. Among those who had physicians, rates were highest among those enrolled in FHTs (64.6%), and lowest for those not in a group. For all groups, screening rates were higher than 60% for those enrolled in FHTs and FHO/FHNs, with the exception of South Asian women and Eastern European and Central Asian women (see Fig. 3). Although having internationally trained physicians significantly lowered chances of screening compared to having domestically trained physicians for the overall cohort, the differences were only significant for women from Caribbean and Latin American (63.6% [CI 62.4–64.8%] vs. 66.1% [CI 65.2–67.0%]) and East Asia and Pacific (61.4% [CI 60.5–62.3%] vs. 64.1% [CI 63.5–64.8%]). Having a family physician who was from the same region as the woman significantly increased the chances of screening for South Asians (50.7% [CI 49.7–51.7%] vs. 46.4% [CI 45.4–47.4%]), Eastern Europe and Central Asian (55.5% [CI 54.2–56.8%] vs. 50.8% [CI 49.8–51.8%]), and Middle East and North Africa (61.4% [CI 59.8 63.0%] vs. 58.0% [CI 56.5–59.5%]).

**Table 4:** Screening rates for immigrant women (CIC) in the study population who lived in Ontario's metropolitan areas for the study period 1 April 2010 to 31 March 2012, by healthcare‐related characteristics and region of origin

Value	Caribbean and Latin America		East Asia and Pacific		Eastern Europe and Central Asia		Middle East and North Africa	
	*N*	%	*N*	%	*N*	%	*N*	%
RUBs (categorized)								
0–1	242	15.3	538	11.2	310	12.2	100	9.2
2	1169	48.7	3385	50.3	1303	38.6	454	42.9
3	11,748	67.5	22,699	68.2	10,086	57.6	4863	64.2
4+	4721	70.7	5590	70.6	3697	62.8	2348	70.5
ADG (categorized)								
0	49	4.4	97	2.8	75	4.2	22	2.6
1–5	5749	56.0	12,925	55.9	5496	44.9	2141	50.3
6–9	7601	71.5	14,171	72.5	6538	62.4	3294	68.0
10+	4481	74.1	5019	75.7	3287	68.2	2308	74.0
Enrollment model								
FHG/CCM	9611	65.5	21,606	64.1	7494	55.1	4480	61.5
FHO/FHN	4909	67.9	5888	63.0	4326	58.7	2193	64.8
FHT	1390	69.1	1771	65.7	1447	60.9	445	66.6
No care	213	21.5	143	7.5	134	9.1	55	9.2
No group	1704	55.4	2551	53.8	1954	44.1	579	52.6
Other^c^	53	58.9	253	72.1	41	51.9	14	45.2
Physician training								
Domestic	11,496	66.1	20,749	64.1	6300	54.4	3295	63.4
International	6034	63.6	11,251	61.4	8858	55.1	4377	60.8
Missing	350	29.5	212	10.5	238	14.3	93	14.1
Physician from same region of origin								
No	16,203	63.9	24,021	60.7	9404	50.8	4360	58.0
Yes	1677	61.5	8191	62.3	5992	55.5	3405	61.4

Value	South Asia		Sub‐Saharan Africa		USA, Australia, and New Zealand		Western Europe	
	*N*	%	*N*	%	*N*	%	*N*	%
RUBs^a^ (categorized)								
0–1	149	5.3	76	12.4	95	17.7	218	21.8
2	1128	35.2	344	46.2	262	51.0	696	54.5
3	13,004	52.5	2826	60.8	1360	64.2	3826	67.4
4+	4519	56.9	1095	61.8	519	69.8	1302	73.3
ADG^b^ (categorized)								
0	36	1.6	24	5.2	33	8.1	59	9.0
1–5	5893	41.3	1448	51.2	965	55.2	2557	57.7
6–9	8594	56.5	1836	63.7	836	70.1	2398	73.3
10+	4277	61.2	1033	64.6	402	70.9	1028	75.3
Enrollment model								
FHG/CCM	13,000	50.2	2238	56.9	614	60.1	1944	62.6
FHO/FHN	3435	55.3	1268	60.5	799	62.0	2193	67.4
FHT	781	57.7	420	66.4	592	64.1	1208	67.0
No care	85	5.3	83	18.6	42	13.3	70	14.8
No group	1426	40.1	301	48.5	95	41.7	545	56.4
Other	73	65.2	32	71.1	94	69.6	82	64.6
Physician training								
Domestic	6254	51.3	2624	58.3	1844	61.4	4373	64.9
International	12,399	50.1	1616	57.9	335	59.6	1557	63.5
Missing	147	8.3	101	20.8	57	16.4	112	20.6
Physician from same region of origin								
No	9017	46.4	3915	55.4	2216	57.1	5337	62.1
Yes	9783	50.7	426	60.3	20	60.6	705	62.4

CIC, Citizenship and Immigration Canada; FHG, Family Health Group; CCM, Comprehensive Care Model; FHO, Family Health Organizations; FHN, Family Health Networks; FHT, Family Health Team.

a RUB = resource utilization bands are part of the Johns Hopkins Adjusted Clinical Group^®^ (ACG^®^) Case Mix System. The RUBs are used to categorize patients based on their expected use of healthcare resources and range from 0 (lowest expected healthcare costs) to 5 (highest expected healthcare costs).

b ADG = aggregated diagnosis groups are part of the Johns Hopkins Adjusted Clinical Group^®^ (ACG^®^) Case Mix system. The ADGs are used to measure the level of comorbidity and range from 0 (no diagnosis group) to 32 (32 distinct diagnosis groups).

c Other = other primary care enrollment models, for example, Rural Northern Physician Group Agreement and Community Health Centres.

#### Screening rates by length of stay in Canada

Comparison of screening rates of immigrant women by region of origin, length of stay (established, recent and new immigrants) in Canada, and sociodemographic, immigration, and health‐related characteristics showed some interesting patterns (data not shown). Sub‐Saharan African who were established immigrants showed a clear income gradient in screening (47.9% for those in lowest income neighborhoods vs. 70.1% in highest income ones). However, among new immigrants in this group, the gradient was much smaller and not significant. Among South Asian established immigrants the gradient was also significant but much smaller (49% for low‐income vs. 57% for high‐income neighborhoods). Rates for new South Asian women were consistently low irrespective of neighborhood income.

Examination of screening rates in relation to health‐related characteristics by length of stay showed that rates of screening increased with increasing number of comorbidities and healthcare resource utilization regardless of length of stay (data not shown). Having a female physician was consistently associated with higher screening rates among all groups regardless of length of stay, except new and recent immigrants from USA, Australia, and New Zealand where physician gender was not significant.

#### Regression modeling

**Figure 4:**
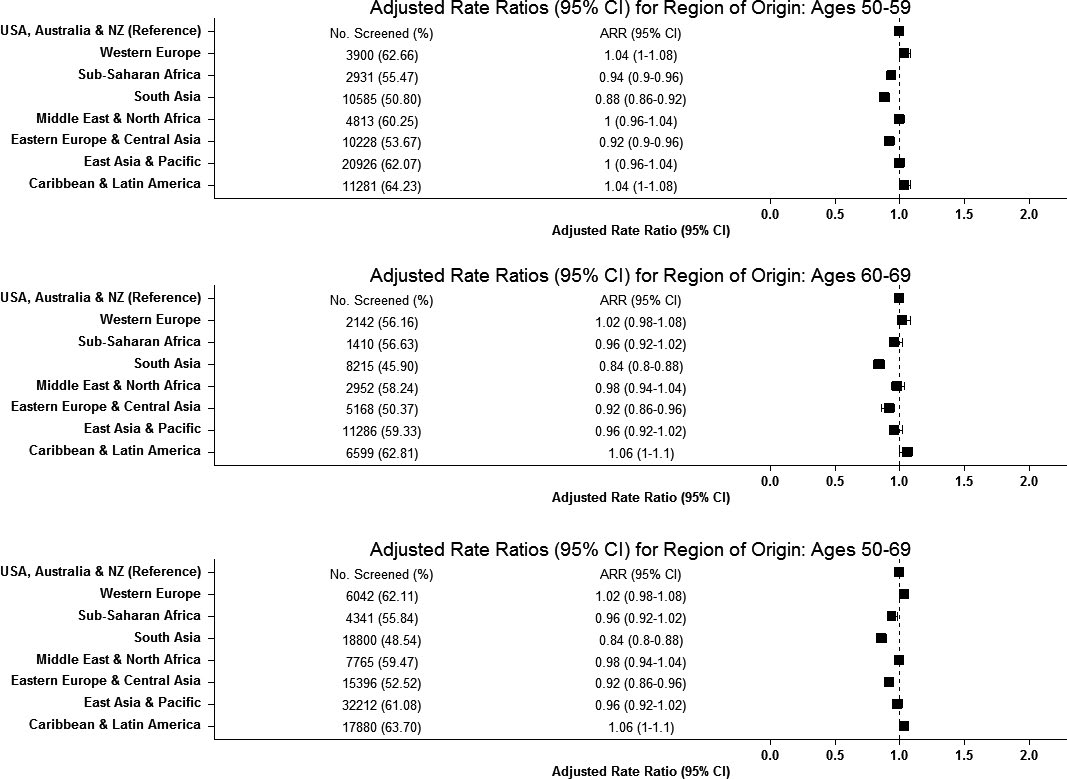
Adjusted rate ratios for appropriate breast cancer screening rates for identified immigrant women in the study, by age group and region of origin.

Figure 4 shows adjusted rate ratios (ARRs) comparing appropriate breast cancer screening rates for women from different origins compared to those from USA, Australia, and New Zealand, by age group. In both the younger (50–59) and older (60–69) age groups, South Asian and Eastern Europe and Central Asian women had significantly lower rates compared to the USA group, while Sub‐Saharan women were only significantly different in the younger age group.

Table 5 shows ARR (with 95% confidence intervals) for risk of screening for identified immigrant women in the study aged 50–69, by region of origin. (NB: women from USA, Australia, and New Zealand were not shown in this table as the model did not converge therefore reliable estimates could not be obtained.) Variables that were significantly associated with low rates of breast cancer screening for all or most of the world regions included living in low income neighborhoods, being admitted as refugees, being new or recent immigrants, not having a general physical examination, not being enrolled in a physician enrollment model, having a male physician, and having an internationally trained physician.

**Table 5:** Adjusted relative risk (with 95% confidence intervals) for risk of screening for identified immigrant women in the study aged 50–69, by region of origin (this table does not include USA, Australia, and NZ)

Value	Caribbean and Latin America	East Asia and Pacific	Eastern Europe and Central Asia	Middle East and North Africa	South Asia	Sub‐Saharan Africa	Western Europe
Sociodemographic factors							
Income quintile							
1	0.96 (0.92–1)	0.96 (0.94–0.98)	0.92 (0.9–0.96)	0.92 (0.88–0.96)	0.92 (0.88–0.96)	0.88 (0.84–0.94)	0.94 (0.9–1)
2	0.96 (0.94–1)	0.98 (0.96–1)	0.94 (0.92–0.98)	0.98 (0.94–1.02)	0.96 (0.92–1)	0.94 (0.88–1)	0.98 (0.94–1.04)
3	0.98 (0.94–1)	1 (0.96–1.02)	0.98 (0.94–1.02)	0.98 (0.94–1)	0.98 (0.94–1.02)	0.96 (0.9–1.02)	1.02 (0.96–1.06)
4	1 (0.96–1.04)	1 (0.98–1.02)	1 (0.96–1.02)	0.98 (0.94–1)	0.98 (0.94–1.02)	0.98 (0.92–1.04)	1.02 (0.98–1.06)
Missing	0.98 (0.74–1.28)	1.06 (0.98–1.16)	1.02 (0.78–1.3)	0.84 (0.54–1.28)	0.84 (0.6–1.18)	1.2 (0.8–1.8)	0.6 (0.26–1.4)
5	1.0	1.0	1.0	1.0	1.0	1.0	1.0
Rurality							
N	1.0	1.0	1.0	1.0	1.0	1.0	1.0
Missing	1.04 (0.56–1.94)	0.98 (0.74–1.3)	0.3 (0.06–1.54)	0.72 (0.12–4.44)	1.54 (0.86–2.72)	0.66 (0.14–2.92)	1.92 (0.66–5.52)
Y	1.02 (0.92–1.12)	1 (0.9–1.12)	0.94 (0.86–1.04)	1 (0.74–1.36)	0.98 (0.78–1.22)	1.14 (0.96–1.34)	1.06 (1–1.1)
Immigration‐related factors							
Immigration class							
Economic	1.0	1.0	1.0	1.0	1.0	1.0	1.0
Family	1 (0.98–1)	0.94 (0.94–0.96)	1 (0.96–1.02)	0.94 (0.92–0.98)	0.9 (0.88–0.92)	0.9 (0.86–0.96)	0.96 (0.92–1)
Other	1.06 (1–1.12)	0.96 (0.92–1)	0.96 (0.88–1.04)	0.96 (0.86–1.06)	0.9 (0.84–0.98)	0.84 (0.74–0.98)	0.86 (0.74–1)
Refugees	1.02 (0.98–1.04)	0.98 (0.94–1)	0.94 (0.92–0.98)	0.92 (0.88–0.94)	0.94 (0.9–0.96)	0.84 (0.8–0.88)	0.8 (0.72–0.9)
Length of stay							
Established	1.0	1.0	1.0	1.0	1.0	1.0	1.0
New	0.94 (0.9–0.98)	0.9 (0.88–0.94)	0.92 (0.86–0.96)	0.9 (0.86–0.96)	0.88 (0.86–0.92)	1.04 (0.96–1.12)	1.06 (0.98–1.14)
Recent	0.98 (0.96–1.02)	0.96 (0.94–0.98)	0.98 (0.94–1.02)	0.96 (0.92–1)	0.92 (0.9–0.94)	0.98 (0.94–1.04)	0.94 (0.88–1)
Healthcare‐related factors							
RUBs							
0–1	1.0	1.0	1.0	1.0	1.0	1.0	1.0
2	1.94 (1.72–2.2)	2.52 (2.26–2.8)	1.9 (1.7–2.16)	2.8 (2.18–3.64)	3.02 (2.54–3.6)	2.14 (1.7–2.7)	1.68 (1.48–1.9)
3	2.48 (2.2–2.78)	3.08 (2.78–3.44)	2.52 (2.24–2.84)	3.86 (2.96–5.04)	3.94 (3.32–4.7)	2.62 (2.1–3.28)	1.94 (1.72–2.18)
4+	2.52 (2.24–2.84)	3.04 (2.74–3.4)	2.58 (2.28–2.92)	3.98 (3.06–5.18)	4.02 (3.38–4.78)	2.66 (2.12–3.34)	2.08 (1.84–2.34)
A003 visit (physician general assessment)							
Visit	1.0	1.0	1.0	1.0	1.0	1.0	1.0
No visit	0.64 (0.62–0.66)	0.56 (0.54–0.58)	0.62 (0.58–0.64)	0.64 (0.62–0.68)	0.54 (0.52–0.56)	0.62 (0.58–0.64)	0.62 (0.6–0.64)
GP visits							
Visit	1 (1–1)	1.02 (1.02–1.02)	1.02 (1–1.02)	1.02 (1–1.02)	1.02 (1.02–1.02)	1 (1–1)	1 (1–1.02)
Enrollment model							
FHT	1.0	1.0	1.0	1.0	1.0	1.0	1.0
FHG/CCM	0.92 (0.88–0.94)	0.86 (0.84–0.9)	0.82 (0.78–0.84)	0.88 (0.84–0.94)	0.82 (0.78–0.88)	0.86 (0.8–0.92)	0.86 (0.82–0.9)
FHO/FHN	0.98 (0.94–1.02)	0.92 (0.88–0.96)	0.92 (0.88–0.96)	0.96 (0.92–1.02)	0.94 (0.88–0.98)	0.98 (0.92–1.04)	0.94 (0.92–0.98)
No group	0.78 (0.74–0.84)	0.72 (0.66–0.76)	0.68 (0.62–0.72)	0.8 (0.74–0.88)	0.7 (0.64–0.76)	0.82 (0.74–0.9)	0.78 (0.72–0.84)
Other	0.88 (0.74–1.04)	1.06 (0.96–1.2)	0.88 (0.74–1.06)	0.68 (0.48–0.96)	1.16 (1–1.34)	1.18 (0.96–1.46)	1 (0.88–1.14)
Physician sex							
Female	1.0	1.0	1.0	1.0	1.0	1.0	1.0
Male	0.94 (0.92–0.96)	0.94 (0.92–0.96)	0.92 (0.88–0.94)	0.9 (0.88–0.94)	0.92 (0.88–0.94)	0.94 (0.9–0.98)	0.94 (0.92–0.98)
Physician training							
Domestic	1.0	1.0	1.0	1.0	1.0	1.0	1.0
International	0.96 (0.94–1)	0.94 (0.92–0.98)	0.98 (0.96–1.02)	0.92 (0.9–0.96)	0.96 (0.94–0.98)	1 (0.96–1.04)	0.98 (0.94–1.02)
Missing	1.1 (0.94–1.26)	1.16 (0.9–1.52)	1.06 (0.92–1.24)	1.06 (0.88–1.28)	0.76 (0.56–1.02)	0.84 (0.48–1.44)	0.62 (0.34–1.18)

RUB, resource utilization bands; FHT, Family Health Team; FHG, Family Health Group; CCM, Comprehensive Care Model; FHO, Family Health Organizations; FHN, Family Health Networks.

Among the health‐related characteristics, not having a general physical examination was associated with the highest risk of not being screened, with ARR values ranging from 0.54 (95% CI 0.52–0.56) for South Asian women to 0.64 (95% CI 0.62–0.66) for Caribbean and Latin American women and 0.64 (95% CI 0.62–0.68) for Middle East and North African women. With respect to physician enrollment models, not being enrolled in any model had the highest risk of not being screened compared to enrollment in FHTs for all regions: ARRs ranged from 0.68 (95% CI 0.62–0.72) among women from Eastern Europe and Central Asia to 0.82 (95% CI 0.74–0.9) among Sub‐Saharan women. Having a male physician decreased the risk of screening for all regions, ARR value was lowest for Middle Eastern and North African women (0.9 [95% CI 0.88–0.94]). Having an internationally trained physician (for women from the East Asian and Pacific, Middle East and North Africa, and South Asia) also decreased risk of screening with the lowest rates among the Middle East group (ARR 0.92 [95% CI 0.9–0.96]). Risk of being screened increased with increasing use of healthcare resources and the gradient was largest among South Asian women: those with four or more RUBs were four times more likely (ARR 4.02 [95% CI 3.38–4.78]) to be screened compared to those with 0–1 RUBs.

For sociodemographic characteristics—living in low‐income neighborhoods was significantly associated with lower screening rates compared to highest income areas for all regions except Western Europe and Caribbean and Latin America, and ARRs were lowest for Sub‐Saharan women (0.88 [95% CI 0.84–0.94]. Entering Canada through Refugee class decreased the risk of screening compared to Economic class for all groups except Caribbean and Latin America and East Asia and Pacific. ARRs for refugees were lowest for Western European women (0.8 [95% CI 0.72–0.9]) and Sub‐Saharan women (0.84 [95% CI 0.8–0.88]). With respect to length of stay in Canada, among new immigrants, South Asians had the lowest risk of being screened (ARR 0.88 [95% CI 0.86–0.92]), while women from Western Europe had the highest risk (ARR 1.06 [0.98–1.14]) compared to established immigrants. Among recent immigrants, women from South Asia (ARR 0.92 [95% CI 0.9–0.94]) and East Asia and Pacific ARR 0.96 [95% CI 0.94–0.98]) had significantly lower risk of being screened compared to their established counterparts. The rest were not significantly different from established counterparts.

## Discussion

In this study, we have shown that despite similarities among immigrant women regarding their low breast cancer screening utilization there were significant differences in their patterns of utilization and access to breast cancer screening services. Overall, immigrant women had slightly lower screening rates (57%) than the province as a whole (61%) 3. These rates are lower than the national and Cancer Care Ontario target of 70% 3, 4. The rate of screening significantly varied by the region of origin with *South Asian* women having the lowest overall rate of utilization, and with women from the Caribbean, Latin America, and Western Europe having screening rates higher than the provincial rate. The results showed that immigrant women are affected by a confluence of sociodemographic and immigration‐related determinants of breast cancer screening and the impact of these factors varied across region of origin. While living in low‐income neighborhoods was independently associated with lower rates of screening across all regions, the gradient was highest among *Sub‐Saharan* women indicating that some ethno‐cultural groups are more impacted by low‐income than others. With the exception of those from the Caribbean and Latin America, immigrant class was associated with breast cancer screening, with overall lower rates observed for women from most groups who entered Canada under *Refugee and Family classes*. Furthermore, screening rates varied by length of stay in Canada: *new migrants* (*being in Canada 5 years or less*), generally had the lowest rates of screening compared to their counterparts who had stayed in Canada longer. Interestingly, those immigrant women who had been in Canada more than 5 years continued to have significant differences in screening rates, suggesting that a longer period of stay in Canada does not guarantee elimination of sociodemographic and structural barriers to screening.

Our findings are analogous to those observed in other Canadian and international studies targeting screening disparities by region of origin and income level 6, 18, 19. Previous studies attributed low rates of breast cancer screening in low‐income populations to structural and individual barriers such as lack of time, transportation issues, hours of operation of mammography centers, and limited knowledge and lack of awareness about available cancer screening 4, 6, 11, 12, 13, 14, 19, 24, 25, 26, 27, 28, 29. Studies exploring breast cancer among refugees and immigrants have also demonstrated low rates among these groups 30, 31, 32 and identified similar barriers like limited knowledge of breast cancer and screening, cultural beliefs, language difficulties, and psychosocial barriers in accessing cancer screening services. Likewise, empirical evidence suggests screening inequities based on length of stay in host countries and attributes this again to individual and structural barriers such as language barrier, cultural and religious beliefs, limited income, and lack of time 7, 8. Percac‐Lima and colleagues 30 demonstrated that barriers to screening among Bosnian refugees and immigrants in the United States could be overcome by using a culturally tailored, language‐concordant navigator program. Similarly, Shirazi et al. 32 proposed a socially, culturally, and religiously tailored community‐based health education program for Muslim Afghan immigrants.

Screening differences were also observed based on enrollment in primary healthcare models, comorbidities, and use of healthcare services across different regions of origin. Not having a regular physical assessment emerged as the screening barrier of most importance for all cultural groups, with the South Asian group being most affected and those from the Middle East and North African the least. Having less contact with the healthcare system (0–1 RUBs), not being enrolled in PEMs, having a male family doctor, or having an internationally trained physician were also independently associated with lower rates of screening for immigrant women across most or all regions of origin. The above‐mentioned factors have been reported to increase the risk of underscreening among women 18, 33, 34, 35, 36, 37. Having a regular checkup provides an opportunity for physicians to educate women on breast cancer and screening, and refer women for appropriate breast cancer screening. Hyman et al. examined physician likelihood of referring women for a mammography and found that insufficient time for patient education was related to lower chance of referral for mammography. Other barriers included women's modesty and religious beliefs, fear of discomfort during mammography, and physicians' forgetting to make an appropriate referral 34, 35. Several studies have shown that being enrolled in FHTs or FHOs increases the chance of cancer screening 18, 35, however, the reason for these findings is not clear 38. Team‐based care is emphasized in FHTs, which means that healthcare practitioners such as nurse practitioners may be playing larger roles. These results suggest that enrollment in primary care models should be explored, and that ensuring access to regular primary care providers, including access to female staff (whether physician, nurse, or nurse practitioner) should be emphasized. It is also imperative to encourage and educate all physicians, but particularly male and internationally trained primary care providers, about breast cancer screening guidelines and the identified risk factors when encountering screening‐eligible women in their practices. The provision of cultural competency training to primary care providers could benefit them in their health communication concerning breast health.

Our study showed that having a family physician who was from the same region as the woman significantly increased the chances of being screened. Particularly, for three regions with overall screening rates lower than others (i.e., South Asians, Eastern Europeans and Central Asians, and East and North Africans). It is possible that physicians from the same origin/ethnic group may have been able to overcome the language barriers to care and had a better understanding of women's behavioral and cultural norms and barriers that they might face in a new country. Empirical evidence on the value of ethnic matching on patients' health outcomes remains inconclusive. However, our study corroborates findings from studies that have found benefits (e.g., improved quality of care, satisfaction, and continuance with care) in patient–physician ethnic concordance during health encounters 39, 40, 41. The fact that linguistically and culturally tailored patient navigation strategies have been shown to be effective in promoting breast cancer screening among minority immigrant and refugees 30, 42, 43, 44, 45 further supports the importance of concordance on factors associated with ethnicity like language, cultural history, and religion in healthcare encounters.

The pattern of breast cancer screening utilization by immigrant women observed in our study is similar to those reported for other types of cancer screening. Lofters and colleagues examined predictors of low cervical cancer screening among immigrant women groups in Ontario and showed that residence in lowest income neighborhoods, not being in a primary care PEM and having a male physician were significantly associated with low cervical cancer screening. In addition, they found that South Asian immigrant women had lower rates of screening and Sub‐Saharan immigrant women were most impacted by neighborhood income disparities 35. An earlier study by Xiong et al. 36 also demonstrated similar findings among Asian Canadian immigrant women. These findings point to the need for a holistic and culturally appropriate approach in promotion of cancer screening in general rather than focusing on a particular cancer.

### Study limitations and strengths

There are a few limitations that should be taken into consideration when interpreting our results. First, the use of cross‐sectional administrative data limits the ability to address causation or account for some confounders such as religion and linguistic abilities that may affect women's participation in screening. Second, the CIC database may not include all immigrants to Ontario particularly those who migrated from other Canadian provinces 15, or those who do not have OHIP coverage. The latter group is likely to have even lower rates of screening compared to the study group. Third, CIC database does not capture any information on nonimmigrant women which limits the exploration of disparities in screening practices between immigrant and nonimmigrant women. However, it does allow to examine disparities in screening practices by immigrant women's region of origin which is consistent with the purpose of this study. Fourth, the use of neighborhood income, an ecological variable, as a proxy for women's income, may not truly reflect the socioeconomic status of all women in a particular area. Fifth, immigrants' region of origin was based on their country of birth which may not be the best indicator for cultural origin and geographic regions of the world may be too broad to allow the emergence of cultural differences among the categories. However, despite these limitations, several factors contribute to the strength of our study, including being a population‐based study with a large sample size using objective instead of self‐report data, and containing all women aged 50–69 with health coverage. In addition, the examination of multiple individual and system‐related variables among the women in individual regions of origin facilitated the identification of subgroups at higher risk that can be targeted in addressing low breast cancer screening rates. Furthermore, the ability to compare our results to those from other cancer screening studies allows the identification of high‐risk groups like South Asian women that can be targeted for multiple cancer screening conditions.

## Conclusions

Results from this study confirm several factors that have been highlighted in other studies and add to the available knowledge. Our study is among the first in Ontario that has explored and been able to demonstrate significant breast cancer disparities based on income, immigration class, length of stay, and world region of origin among immigrant women. In addition, breast cancer screening inequities existed based on primary care enrollment models, healthcare utilization, and primary care provider's gender and training. Multiple interventions entailing cross‐sector collaboration are required to address the cancer screening inequities among immigrant women in Ontario. Increasing access to primary care and physician services is a crucial component. Efforts need to be made to increase immigrant women's access to regular primary care providers through programs such as the Ontario Health Care Connect program. This can be done in collaboration with settlement and community agencies that serve the immigrant groups. Outreach should be done to identify and connect new comers and recent immigrants. Physician characteristics play an important role in increasing cancer screening rates. Strategies in this area include education of physicians, particularly internationally trained primary care providers, on identified risk factors when seeing screening‐eligible women in their practices. The health system should endeavor to increase access to female healthcare providers for the majority of women, and increase immigrant women enrollment in physician enrollment models. Culturally appropriate public education campaigns are also required to increase immigrant women's awareness of the benefits of breast cancer screening. Targeted programs are also required that focus on identifying and addressing barriers for particular subsets of ethnic groups such as South Asian women. The congruency of our findings related to South Asian and Sub‐Saharan women with those from cervical screening studies supports the need for first identifying barriers and then designing screening programs for high‐risk subgroups of women that involves culturally tailored patient‐centered programs that lean toward multipronged cancer screening rather than individual cancers.

## Conflict of Interest

None declared.
